# Polymer Micro/Nanofabrication and Manufacturing

**DOI:** 10.3390/polym15061350

**Published:** 2023-03-08

**Authors:** Yi-Je Juang

**Affiliations:** 1 Department of Chemical Engineering, National Cheng Kung University, No. 1 University Road, Tainan 70101, Taiwan; yjjuang@mail.ncku.edu.tw; 2 Core Facility Center, National Cheng Kung University, No. 1 University Road, Tainan 70101, Taiwan; 3 Research Center for Energy Technology and Strategy, National Cheng Kung University, No. 1 University Road, Tainan 70101, Taiwan

Polymer microfabrication/nanofabrication and manufacturing are processes that involve the creation of small-scale structures using various polymeric materials. This technique has gained significant attention since the 1980s due to its ability to produce precise and complex structures with high efficiency and cost-effectiveness. The resulting structures can be found in a wide range of applications such as microfluidics, biosensors, microelectronics, micro-optics, and tissue engineering. In this Special Issue, Juang and Chiu reviewed the fabrication techniques for polymer microfluidics, which can be categorized into the mold-based and non-mold-based approaches. Various techniques such as micro-embossing, micro-injection molding, casting, CNC micromachining, laser micromachining, and 3D printing are discussed [1]. For the mold-based approaches, Zhu et al. investigated the fabrication of polymer microstructures via ultrasonic-assisted embossing and it was found that the embossing time was less than a few seconds and more than a 75% average filling rate was achieved [2]. Juang et al. extended the application of micro-embossing to fabricate microfluidic paper-based analytical devices (µPADs) [3]. By utilizing the one-step strategy, i.e., forming the protruded channel and sealing the backside of the channel simultaneously, the processing time was reduced to around 5 s and the µPADs as fabricated were used for glucose detection with a linear relationship between 5 and 50 mM. Numerical simulation was conducted to address the issues in micro-injection molding. Wu et al. utilized the improved non-dominated sorting genetic algorithm NSGA-II for the optimization of micro-injection-molded gear shrinkage [4]. The optimization results of the NSGA-II algorithm were verified using Moldflow simulation and the accuracy of the optimized method was further compared with the experimental results. It was found that the tooth profile accuracy of the micro-injection-molded gears was improved. The finite element simulation was also applied to the acoustic streaming and mixing characteristics in ultrasonic plasticization micro-injection molding (UPMIM) [5]. The authors found that several melt vortices were developed in the plasticizing chamber via ultrasonic vibrations, with the melt rotating around the center of the vortex. Moreover, the Stokes drag force acting on the fluorescent particles was two orders of magnitude greater than the acoustic radiation force. As for the non-mold-based approaches, Chen et al. utilized the FDM 3D printer and stereolithographic printer to construct a lifelike brain glioblastoma simulator [6] and a simulator containing the brain stem, soft brain tissue, carotid arteries, and a hollow transparent circle of Willis [7] for the training of neurosurgeons. They also exploited digital light processing (DLP) stereolithographic printing to fabricate microfluidic devices with an extremely high aspect ratio equal to 40 [8]. Lai and Yu designed the ink for 3D printable sensors with cationic cellulose nanocrystals (CCNCs) and zwitterionic hydrogels [9]. It was found that the nanocomposite hydrogels made by the designed ink possess a stronger physical network at lower nanofiller concentrations. As a result, they showed good mechanical strength, high transparency, and 3D printability. Examples were also demonstrated by applying various polymer microfabrication techniques such as ion-milling on epoxy resin [10], the fabrication of oblique structures via hard X-ray lithography [11], paper-based microfluidics constructed via spraying [12], micromechanical punch for the fabrication of non-spherical microparticles [13], electrostatic self-assembly of composite nanofiber yarn [14], and mechanical and chemical polishing of the surface of polymer microchannels [15]. The editors are confident that the readers will benefit from this book by gaining common knowledge regarding polymer microfabrication techniques and a better understanding of the practical uses and versatility of these techniques through the demonstrated examples. This book is also a useful resource for the general audience who are interested in polymer microfabrication and would like to embark upon their exploration into this field.
